# The Role of B7 Family Molecules in Maternal–Fetal Immunity

**DOI:** 10.3389/fimmu.2020.00458

**Published:** 2020-03-24

**Authors:** Yongbo Zhao, Qingliang Zheng, Liping Jin

**Affiliations:** Clinical and Translational Research Center of Shanghai First Maternity and Infant Hospital, Tongji University School of Medicine, Shanghai, China

**Keywords:** B7 family, costimulatory signal, coinhibitory signal, maternal–fetal immunity, maternal–fetal interface, reproductive immunity

## Abstract

Pregnancy is a complex but well-arranged process, and a healthy fetus requires immune privilege and surveillance in the presence of paternally derived antigens. Maternal and fetal cells interact at the maternal–fetal interface. The upregulation and downregulation of maternal immunity executed by the leukocyte population predominantly depend on the activity of decidual natural killer cells and trophoblasts and are further modulated by a series of duplex signals. The B7 family, which consists of B7-1, B7-2, B7-H1, B7-DC, B7-H2, B7-H3, B7-H4, B7-H5, BTNL2, B7-H6, and B7-H7, is one of the most characterized and widely distributed signaling molecule superfamilies and conducts both stimulatory and inhibitory signals through separate interactions. In particular, the roles of B7-1, B7-2, B7-H1, and their corresponding receptors in the progression of normal pregnancy and some pregnancy complications have been extensively studied. Together with the TCR–MHC complex, B7 and its receptors play a critical role in cell proliferation and cytokine secretion. Depending on this ligand–receptor crosstalk, the balance between the tolerance and rejection of the fetus is perfectly maintained. This review aims to provide an overview of the current knowledge of the B7 family and its functions in regulating maternal–fetal immunity through individual interactions.

## Introduction

**FIGURE 1:**
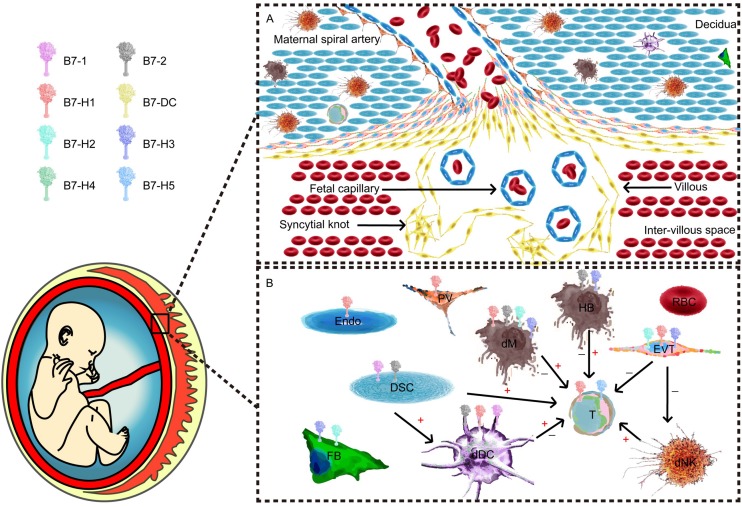
Maternal–fetal interface and major component cells. **(A)** Cellular components of the maternal–fetal interface throughout all pregnancy trimesters. **(B)** B7 expression and cellular interactions. T cells and their subpopulations are recognized as one of the most important effector cells during maternal–fetal immunity. Different antigen-presenting cells (APCs) and trophoblasts play unique roles in the network.

Pregnancy is a complex but well-orchestrated process, and studies conducted in mice have indicated that the maternal immune system is exposed to paternally inherited fetal antigens as early as at the time of insemination. Once the placenta fully matures, this exposure wanes until the establishment of a uterine blood supply to the placenta and then becomes robust again (1). The fetus is genetically dissimilar and thus immunologically incompatible (2). This immunological paradox suggests a fully efficient mechanism of immunosuppression. Maternal–fetal immunity in the decidua requires all cellular elements of the mucosa, namely, stromal, glandular, and immune cells (Figure 1), and the set of immune cells prominently involves decidual natural killer cells, macrophages and regulatory T cells. Mechanistic research has attempted to reveal how the fetus and placenta establish tolerance to the maternal immune system, including the suppression of overreactive immune cells and the secretion of locally distributed soluble products. Recent studies have indicated an important role for immunomodulators in facilitating maternal T-cell tolerance to fetal antigens.

Cosignaling molecules are expressed on the cell surface and transduce positive (costimulatory) and negative (coinhibitory) signals. After an antigen is recognized by a T-cell receptor, a cosignaling molecule, or second signal, is necessary for T-cell activation. In the fields of cancer, transplantation, and autoimmune disease, these cosignaling molecules have been vigorously studied, and some molecules have been identified as immune checkpoints. The B7 family is one of the most characterized cosignaling network superfamilies and plays an important role in the modification of T-cell activation and tolerance (3). Numerous studies have attempted to delineate the underlying pathways using various techniques, such as non-specific suppression and cell surface-related stimulation.

This review aims to provide an overview of the current knowledge of the B7 family and its functions in regulating maternal–fetal immunity through individual ligand–receptor complexes. It also highlights the potency of the *in vitro* blockage of specific members of the B7 family for miscarriage immunotherapy.

## A Two-Signal Model of T-Cell Activation

**FIGURE 2:**
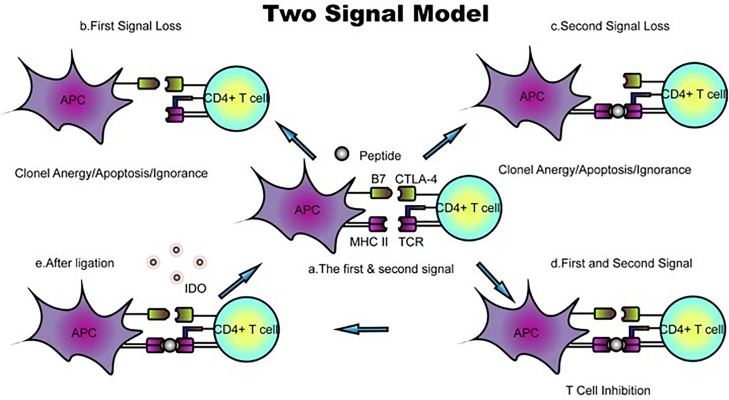
Classic two-signal model for T-cell activation/inhibition. **(a)** The first signal consists of peptides or antigens presented by the TCR–MHC complex, whereas B7 family members and their receptors function as the second signal. **(b,c)** Loss of either the first or second signal prevents activation and leads to T-cell clone anergy/apoptosis/ignorance. **(d)** In the presence of TCR–MHC and B7 ligand–receptor interactions, positive or negative signals are transduced. The ligation of B7 with CTLA-4 inhibits T-cell proliferation. **(e)** B7-CTLA-4 ligation upregulates the expression of the reverse signaling enzyme indoleamine 2,3-dioxygenase (IDO), which is believed to be involved in T-cell-induced fetal rejection. DC, dendritic cell; dM, decidual macrophage; dS, decidual stromal cell; Endo, endothelial cell; Epi, epithelial glandular cell; F, fibroblast; HB, Hofbauer cell; PV, perivascular cell; SCT, syncytiotrophoblast; VCT, villous cytotrophoblast; EVT, extravillous trophoblast; RBC, red blood cell.

Because the conventional scheme of the cell surface, MHC fails to explain many features of an allogeneic reaction. Lafferty and Cunningham have suggested a cell interaction model that accounts for this inadequacy based on Bretscher and Cohn’s two-signal model (4). Initially described as “a species−specific proliferation signal,” the cosignaling network was not validated until CD28, and subsequently, its ligand, B7-1, was found to amplify the TCR signal. Owing to the identification of cytotoxic T lymphocyte antigen 4 (CTLA4), a coinhibitory receptor that binds to B7-1, the two-signal model for T-cell activation has been widely accepted. In the classic two-signal model of T-cell activation, signal one consists of engagement of the TCR and the peptide–MHC complex, and signal two arises from cosignaling from antigen-presenting cells (APCs). T-cell responses are initiated only if the two signals independently confer specificity; otherwise, this signaling induces T lymphocyte clonal anergy and unresponsiveness *in vitro* and *in vivo*, respectively (5, 6) (Figure 2). Costimulatory molecules, particularly B7 family members, have evolved into an important aspect of immune regulation.

The discovery of CD28 provides evidence of a costimulatory prototype. CD28, which is localized on human chromosome 2q33 and mouse chromosome IC/1, regulates the T-cell stimulatory signal (7), and this signal is constitutively expressed on the cell surface of naïve CD4^+^ and CD8^+^ T cells. Overall, CD28 provides an essential costimulatory signal for T-cell growth and survival upon ligation by B7-1 and B7-2 on APCs. In addition, in human T-cell lines, CD28 enhances integrin-mediated adhesion and induces cytoskeletal rearrangements through the Rho family GTPases Rac1 and Cdc42 (8, 9). In CD28-deficient mice, primed T cells show inefficient localization to non-lymphoid antigenic sites. Further studies using transgenic mice have highlighted the prominent function of CD28-induced PI3K pathways in regulating T-cell trafficking (10). The role of the CD28/B7 pathway in pregnancy-related complications has not been illustrated. However, a retrospective study analyzed a polymorphism of CD28 and concluded that the SNP of CD28 in recurrent spontaneous abortion (RSA) patients was distinct from that in normal pregnant women (11). A previous human study identified significantly higher numbers of CD28^–^ suppressor T cells at the maternal–fetal interface than in peripheral blood, which suggested an immunomodulatory function for this T-cell subset (12). The literature also provides evidence supporting the participation of CD28 in the process of embryo implantation in mice (13).

CTLA-4 is one of the earliest identified coinhibitory receptors, and this single-V-domain member of the immunoglobulin superfamily shares not only its chromosomal region but also its hinge region (hexamer MYPPPY) to bind to CD80 (B7−1) and CD86 (B7−2) ligands. Compared with CD28, CTLA-4 can be detected only after conventional T-cell activation but is constitutively expressed on regulatory T cells (Tregs) (14). CTLA-4-knockout mice rapidly develop fatal lymphocytic infiltration of multiple organs, which supports the previous view of CTLA-4 as a proliferation inhibitor (15). However, significantly higher levels of soluble CTLA−4 (sCTLA-4) have recently been observed in patients with systemic lupus erythematosus (SLE) compared with normal individuals. Further studies have shown that sCTLA-4 reduces inhibitory signals and promotes T-cell activation by inducing CTLA-4-CD80/CD86 binding (16). The CTLA-4-induced T-cell fate can be either positive or negative. Similar to the results obtained for CD28, polymorphisms of CTLA-4 might influence the risk of developing RSA in humans (17). Increased levels of CTLA-4^+^ cells are detected in patients with repeated implantation failure (RIF), indicating a negative role in regulating endometrium receptivity (18).

## B7-1 and B7-2

B7-1 and B7-2, which are also individually known as CD80 and CD86, are both localized on human chromosome 3 and rat chromosome 11. After these proteins were first cloned in the early 1990s, B7-1/B7-2 was reported as a costimulatory signal to decidual stromal cells (DSCs). Interestingly, B7-1/B7-2 is mainly located on class II human leukocyte antigen (HLA)^+^ predecidual cells (pre-DSCs, also known as DSC precursors) and serves to stimulate allogeneic T cells. Despite their functional similarity and myelomonocytic origin, CD10^+^CD13^+^ pre-DSCs are also involved in different immune functions depending on their differentiation status, such as the secretion of immunosuppressive substances and the production of cytokines (19). These cells are related to dendritic cells (DCs), which are established inducers of T-cell immunity and mediators of T-cell tolerance. Since then, human studies have further characterized B7-1 and B7-2 expression on decidual APCs, including DCs and macrophages. Immature decidual DCs express only small quantities of MHC II and B7-1/B7-2 molecules, leading to clonal T cell anergy (20). These MHC II-high, costimulatory-molecule-high and cytokine-high DCs are considered fully mature DCs (21, 22). CD80 and CD86 are highly expressed on the surface of mature decidual DCs compared with peripheral blood DCs (23). Monocytes from healthy pregnant women differentiate into less phenotypically mature DCs that express lower levels of CD80 and CD86. However, a statistical analysis revealed that decidual tissues obtained from RSA patients exhibit upregulated mRNA and protein levels of CD86 and low CD80 expression (24). Similar conclusions were obtained with an abortion-prone model of CBA/J × DBA/2. The upregulation of CD86 in splenic B cells is observed starting on the third day after mating, and its expression level is even higher on day 14, whereas CD80 is found at a lower expression level (25). The blockade of CD86 signaling in abortion-prone mice improves pregnancy outcomes by facilitating a Th2 bias at the maternal–fetal interface and in expanding peripheral Tregs (26). In other pregnancy complications, such as preeclampsia, peripheral DCs from patients express higher levels of both CD80 and CD86. *In vitro* culture shows a strong ability to promote the differentiation of naïve CD4^+^ T cells into Th1/Th17 cells (27). Following *Toxoplasma gondii* infection, the upregulation of CD80/CD86 on decidual DCs contributes to abnormal pregnancy outcomes (28). These findings indicate that B7-1 and B7-2 assist decidual DCs in maintaining a Th2-dominant state, which is beneficial to a gestational outcome.

B7-1 and B7-2 bind to CTLA-4 with greater affinity than CD28, and therefore, CTLA-4 might play a particular role in the mediation of maternal–fetal immunity (29). Previous studies have proven that the appropriate frequency and function of decidual Tim-3^+^CTLA-4^+^CD8^+^ T cells are important in the maintenance of normal human pregnancy (30). Along with its constitutive and restricted expression on Tregs, CTLA-4 expression is involved in the immune-suppressive function of these cells (31). The ligation of B7-1 and B7-2 with CTLA-4 upregulates a reverse signal by indoleamine 2,3-dioxygenase (IDO). IDO is highly expressed at the human maternal–fetal interface and is capable of inducing T-cell-related fetal rejection (32, 33). In addition to its expression in uterine gland epithelium and leucocytes, most IDO1 is found in vascular endothelial cells and trophoblasts (34). Studies have shown that CTLA-4 is found at lower transcription and translation levels in decidual tissues isolated from RSA patients than in those isolated from normal pregnancies. More specifically, the CTLA-4 polymorphism 49A-G is associated with the development of placental abruption and preeclampsia, and women with the G allele are at risk for these complications (35).

Another contribution of B7-1/B7-2 at the maternal–fetal interface originates from decidual macrophages and placental macrophages of fetal origin (Hofbauer cells, HBCs). Decidual macrophages are the second most abundant immune cells at the maternal–fetal interface and polarize toward the M1 phenotype, which is characterized by the expression of CD80/CD86 and proinflammatory cytokines. Based on the phenotypic features of macrophages from healthy human decidua, the higher expression of CD80 and CD86 in decidual macrophages at early/mid-pregnancy suggests that these cells have a more activated phenotype than that observed at term (36). Decidual macrophages from term pregnancy have low expression levels of CD80 and CD86, which suggests that these cells play a role in the prevention of maternal T-lymphocyte activation. The expression of CD80 and CD86 in macrophages from RSA patients is higher than that in controls, and this abnormal macrophage subset might also influence the immunosuppressive effects of Tregs (37). Villitis of unknown etiology (VUE), chorioamnionitis-induced spontaneous preterm birth, severe preeclampsia, and HELLP syndrome are related to a shift in the macrophage phenotype from M1 to M2, and this shift is accompanied by a change in the phenotypic markers from CD80/CD86 to CD163/CD206 (38, 39). *T. gondii* infection increases the expression of B7-2 on macrophages (40). HBCs have an M2-like profile, accompanied by high CD163 and CD206 expression and the production of IL-10 and TGF-β (41). These CD80^–^CD86^–^ cells play a role in normal human placental development and might be responsible for some infections (41). Despite the general view, recent studies in humans have claimed that gestational diabetes mellitus (GDM) causes temporary M1 proinflammation in HBCs, with a trend for increasing CD86 expression (42). *In vitro* coinfection with human cytomegalovirus (HCMV) and HIV enhances susceptibility and dysregulates CD80 expression in HBCs (43).

## B7-H1 and B7-DC

B7-H1 and B7-DC share the same inhibitory receptor, programmed death 1 (PD-1). Both B7-H1 and B7-DC are type I transmembrane proteins encoded by the adjacent genes CD274 and Pdcd1lg2, respectively, on human chromosome 9 and mouse chromosome 19. Identified as the third member of the B7 family in 2000 (44), B7-H1, an alternative name for CD274 or programmed cell death ligand-1 (PD-L1), has been implicated in the PD-1 immunoinhibitory pathway (45). B7-DC, also called CD273 or PD-L2, was identified as the second ligand of PD-1 in 2001, and its ligation markedly inhibits multiple T-cell responses (46). Observed in both normal pregnant women and murine models, the upregulation of PD-1 on decidual CD4^+^ T cells defines a specific effector/memory subset of CD4^+^ T cells and promotes Th2 bias at the maternal–fetal interface (47). CD8^+^ T cells coexpressing PD-1 are downregulated in the decidua of abortion-prone female mice (48). Several findings indicate that PD-1 pathways play critical roles in the maintenance of normal pregnancy.

B7-H1 is abundantly and constitutively expressed on hemopoietic-like macrophages, DCs, B cells, bone marrow (BM)−derived mast cells, T cells, and non-hemopoietic cells in epithelial, vascular endothelial, pancreatic, and immune−privileged sites, such as the placenta. In a previous study, Petroff M. G. et al. found that trophoblast cells are the source of term placental B7-H1 mRNA, and B7-H1 expression in second- and third-trimester placentas is higher than that in the first trimester. Increased levels of oxygen might be responsible for this elevation (49). Immunohistochemistry demonstrates that in early and term normal human placenta, PD-L1 is highly expressed in syncytiotrophoblasts and at a markedly lower level in intermediate trophoblastic cells located in the chorion laeve and implantation site. PD-L1 immunoreactivity is weak/undetectable in cytotrophoblastic cells (50). However, due to a lack of MHC molecules, the capacity of syncytiotrophoblasts to deliver a reverse signal via PD-1 ligation is prohibited; instead, syncytiotrophoblasts transmit signals to inactivate PD-1-expressing immune cells (49). The epidermal growth factor receptor (EGFR) and JAK2/STAT1 pathways might account for the upregulation of B7-H1 in cytotrophoblasts (51). The maternal blood level of soluble PD-L1 (sPD-L1) is elevated in normal pregnant women at the early stage compared with non-pregnant controls (52). Compared with the findings in peripheral blood, the increased expression of B7-H1 in healthy human decidual CD4^+^ T cell, Treg, NKT-like, and CD56^+^ NK cell subsets is accompanied by elevated PD-1 expression in decidual CD4^+^ T cells, CD8^+^ T cells, and NKT-like cells. Additionally, the cytotoxic potential of decidual PD1/NKG2D double-positive CD8^+^ T cells is significantly decreased. These results indicate that the PD-1/PD-L1 pathway might play a novel role in maintaining the local immunological environment. This immune checkpoint, accompanied by the activation receptor NKG2D, can regulate decidual CD8^+^ Tc cell subsets and contribute to maternal immunotolerance (53). A previous analysis showed that B7-H1 is unique to CD14^+^ decidual macrophages isolated from early pregnancy but is not observed on term or peripheral macrophages. The interaction between macrophages and T cells via B7-H1 ligation to PD-1 remarkably suppresses the production of IFN-γ (54), which suggests an important role in local immune homeostasis.

Since Guleria et al. (55) first reported that the blockade of B7-H1 signaling during murine pregnancy resulted in increased rejection of allogeneic but not syngeneic concepts, many studies have attempted to reveal the underlying molecular mechanism. A study performed by another research group showed that PD-1 signaling blockade did not change the CD4^+^CD25^+^Treg percentage in decidua from RSA patients but increased the CD4^+^ T cell percentage and the level of IFN−γ (56). Because the PD-L1 mRNA level in the decidua is decreased in RSA patients but the PD-1 mRNA level is unchanged, one hypothesis is that the impairment of Treg function in RSA cases might be due to the decrease in PD-L1 in the decidua rather than a change in PD-1. Group 3 innate lymphoid cells (ILC3s) have been detected in both murine and human decidual tissues, where they are relevant to the induction and maintenance of pregnancy. Because ILC3s express PD-1 and intermediate extravillous trophoblasts (iEVTs) express PD-L1, alteration of the PD-1/PD-L1 axis might break immune tolerance, resulting in pregnancy failure (57). The maternal sPD-L1 levels are relatively higher in women with preeclampsia than in normotensive pregnant women (58). Another research group showed a similar elevation of PD-L1 in Tregs from preeclampsia patients, although the percentages of Tregs and Treg-related cytokines, such as TGF-β, IL-10, and IL-3, were decreased. Furthermore, the aberrant differentiation of Tregs can be rectified by the PD-1/PD-L1 activator PD-L1 Fc, which indicates a potential therapeutic value of the PD-1/PD-L1 pathway for patients with preeclampsia (59). In preeclamptic rats, the protective effect of PD-L1 Fc is realized by reversing the Treg/Th17 cell imbalance and placental damage (60). An *in vitro* experiment demonstrated that a splice variant form of PD-L1 (secPD-L1) could inhibit T-cell proliferation. secPD-L1 has been detected in malignant cells but can also be considered a negative immune regulator in pregnancy complications (61). Taken together, these results suggest that the PD-1/B7-H1 checkpoint maintains the local immunological environment by interacting with different immune cells and mediating cytokine secretion.

The expression of B7-DCs was previously recognized as restricted to DCs and strongly costimulates IFN−γ production from isolated naïve T cells (62). B7-DC-PD-1 exhibits higher affinity than B7-H1 for PD-1 but very limited expression compared with B7-H1, and its interactions profoundly inhibit B7-CD28 signals at relatively low antigen concentrations and promote T-cell proliferation. In contrast, these interactions lead to a reduction in cytokine production (63). In human placental tissues, B7-DC is prominent on the apical surface of syncytiotrophoblasts during early pregnancy, and this protein is not present in trophoblasts at term. Detectable B7-DC immunoreactivity is detected in many but not all placental blood vessel endothelial cells at term. In the larger stem villi, smooth muscle cells surrounding arterioles as well as fibroblasts also express B7-DCs. However, B7-DC immunoreactivity is virtually absent in term trophoblast populations (64). Wang G. et al. found that B7-DC is also highly expressed in human placenta mesenchymal stem cells (hPMSCs), which possess strong immunosuppressive properties in healthy pregnancies. PDL knockdown in hPMSCs inhibits T-cell proliferation (65). Higher expression of PD-L2 is observed in human amniotic mesenchymal stromal cells (hAMSCs) from preeclampsia patients than in hAMSCs from normal pregnant women, but conversely, these cells participate in offsetting the inflammatory environment that characterizes preeclampsia. *In vitro* experiments have shown that elevated PD-L2 levels in hAMSCs are associated with inhibition of CD4^+^/CD8^+^ T-cell proliferation by suppressing Th1/Th2/Th17 polarization, inducing Tregs, blocking DCs, and switching M1 to M2 differentiation (66). To summarize, B7-DC is expressed on multiple cells and fluctuates during different pregnancy phases. Both positive and negative signals are conducted depending on the B7-DC concentration and the cell population.

## B7-H2

B7-H2 has different aliases because it was identified by several independent investigators. This molecule is divided into two splice variants designated inducible costimulator ligand (ICOSL)/B7RP-1/CD275 and GL50, which share 19–20% sequence identity with B7-1 and B7-2. A Northern blot analysis by Ling V. et al. indicated that GL50 is expressed on the embryonic yolk sac and in fetal liver samples (67). The presence of GL50 transcripts in embryo-derived yolk sac tissue leads to the intriguing possibility that GL50 might skew the cytokine profiles of maternal T cells toward an immunoprotective Th2 phenotype during pregnancy. However, the available evidence does not support this role of GL50 in maternal–fetal immunity. Initially regarded as a modulator of the tumor microenvironment, B7-H2 binds to ICOS to induce a variety of cytokines and prevents the apoptosis of preactivated human T cells (68). Genetically located on human chromosome 21p12, B7-H2 mRNA is expressed on human cytotrophoblasts in the first trimester, and this protein is also detected on the HUVEC, JEG-3, and Jar cell lines but is undetectable in trophoblast cells. An immunohistochemistry analysis showed specific B7-H2 antibody binding in first-trimester human placentas, extravillous trophoblast cells within cell columns, particularly those cells distal to the villi, and cell islands. Villous trophoblast cells, syncytiotrophoblast cells and stromal cells (most likely macrophages and fibroblasts) show weak B7-H2 immunoreactivity. However, in term placentas, the stromal cells of large stem villi are the predominant B7-H2-expressing cells. In contrast to the very limited expression of B7-H2 in villous cytotrophoblast cells, term extravillous cytotrophoblast cells of the basal plate and chorion membrane show a marked, consistent reaction to the B7-H2 antibody. Macrophages in term placentas strongly express the B7-H2 protein (64). The expression of ICOS, a simulator of T-cell activation, is strongly elevated in healthy pregnant women, whereas its corresponding costimulatory molecule B7-H2 is decreased on monocytes of pregnant women compared with those of non-pregnant women (69). In an allogeneic CBA female × B6 male pregnancy model, blockade of the ICOS-B7-H2 pathway results in increased fetal absorption and a decreased fetal survival rate. After treatment with anti-B7-H2 monoclonal antibody, the levels of regulatory markers, such as IDO and TGF-β1, and CD8^+^ T cells are reduced, whereas the levels of effector cytokines such as IFN−γ are shown as a marked local elevation in the murine placenta. The deleterious effect of B7-H2 blockade is maintained only in CD4-knockout mice and not in CD8-knockout mice, which suggests a crucial role for CD8^+^ T cells (CD8^+^CD103^+^, in particular) in ICOS-B7-H2 axis-related immune mediation. The adoptive transfer of this T cell subset has the ability to abrogate the deleterious effect (70).

Inducible costimulator ligand is the third member of the CD28/CTLA-4 family. In human placenta, strong ICOS expression is observed on CD4^+^ and CD8^+^ decidual T cells but not peripheral T cells. The coculture of decidual T cells with JEG3 cells results in the production of IL-10 and IFN-γ by T cells, and this effect can be blocked by the antibody-specific masking of B7-H2 (71). ICOS expression is also observed on Tregs in the decidua. In an ICOS-deficient model, the Treg cell frequency is significantly reduced, and the production of IFN-γ, IL-6, and TNF-α is enhanced, rendering a Th1-prone immune phenotype (72, 73). Furthermore, the regular differentiation of both ICOS^+^ and ICOS^–^CD45RA^+^CD31^+^ recent thymic emigrant (RTE) Tregs is highly required to ensure a healthy pregnancy course. Disturbed differentiation into CD45RA^–^CD31^–^ memory Tregs is associated with the occurrence of preeclampsia and HELLP syndrome (74). A delayed Th2 immunity defect is observed in ICOS^–/–^ mice, and the function of ICOS to direct T cells toward the Th2 effector state is particularly valued due to the sustained preferential expression of ICOS on Th2-immune cell subsets (75). Although the exact role of ICOS-B7-H2 has not been elucidated, ICOS-dependent P110δ-PI3K isoform activation is emphasized in follicular helper T (TFH) cell development and maintenance. Another study highlighted the function of CXCR3^+^PD-1^+^ICOS^+^ TFH cells and their cytokine profiles, IL-21, IL-6, IL-10, IL-17, and IFN-γ, in avoiding fetal rejection (76). These findings again suggest that T-cell cytokine modulation by ICOS-B7-H2 interactions is important in the delicate immune balance at the maternal–fetal interface.

## B7-H3

Initially discovered as a costimulator of CD4^+^, CD8^+^, and cytotoxic T-cell proliferation as well as an inducer of IFN-γ production, B7-H3 (also known as CD276 or B7RP-2) is considered a coinhibitor of T cells (77, 78). B7-H3, which maps to human chromosome 15q24.1, is a type 1 transmembrane protein located on chromosome 15 and shares 20–27% amino acid identity with other B7 family members (77). Based on the many contradictory roles of B7-H3, TREM-like transcript 2 (TLT-2) might not be the only potential receptor for this protein on the T-cell surface (79). Similar to B7-H2, B7-H3 transcription by cytotrophoblast cells is constant from the first trimester to full term in humans. In first-trimester placenta, extravillous trophoblast cells show striking expression of B7-H3 protein on the cell surface. Villous cytotrophoblast and syncytiotrophoblast cells exhibit only limited B7-H3 expression, but fibroblasts and Hofbauer cells clearly show B7-H3 expression. In term placenta, syncytiotrophoblast cells only exhibit light B7-H3 staining at their apical surface. In contrast, extravillous trophoblast cells of the basal plate and many of those in the chorion membrane robustly express this protein (64). Exosomes purified from first-trimester and term human placental explant cultures carry B7-H3. Additionally, isolated cytotrophoblast cells and syncytiotrophoblasts in culture are capable of secreting exosomes containing B7-H3 (80). Indeed, receptor–ligand interactions appear to affect exosomes on NK and T cells, whereas other researchers have shown that trophoblast-derived microvesicles and exosomes can be engulfed by phagocytes. These findings raise the possibility that B7-H3-containing exosomes serve as vehicular shuttles for paternally inherited placental antigens that are ultimately cross-presented to maternal T cells by APCs. Further studies have shown that B7-H3 translation is restricted to inflammatory cytokine-induced DCs and monocytes (78). In the first trimester, the number of myeloid DCs expressing thymic stromal lymphopoietin receptor (CD11c^+^TSLP-R^+^ cells) and the B7-H3 molecule is decreased after exposure to estriol (81). Because estriol at the typical concentrations detected during pregnancy reduces thymocyte differentiation and inhibits the maturation of invariant T cells with the functions of NK cells that can induce RSA, it can be assumed that the expression of B7-H3 likely plays a role in spontaneous fetal loss and that the balance of immunocompetent cells/molecules is maintained by hormonal control. B7-H3 is now regarded as a new immune checkpoint, and its role in autoimmune diseases and cancer has been widely studied. However, its role in pregnancy-related diseases and at the maternal–fetal interface has not been elucidated. In B7-H3^–/–^ mice, Th1 differentiation is induced in response to more severe airway inflammation, faster experimental autoimmune encephalomyelitis development and higher autoantibody concentrations. In patients who suffer esophageal squamous cell carcinoma, B7-H3 expression is likely to be positively related to the infiltration intensity of Tregs (82). These studies provide support for the development of new therapies for some pregnancy complications characterized by an impaired immunological status. The augmentation of host B7-H3, which promotes Th2 differentiation, Treg induction, T cell suppression, and IFN-γ production, might benefit fetal maintenance. As mentioned above, B7-H3 exerts contradictory effects on T cells. Previous studies have demonstrated that human B7-H3-Ig protein increases the proliferation of CD4^+^ and CD8^+^ T cells and enhances cytotoxic T-cell activity (79). Therefore, the treatment of pregnancy complications by blocking B7-H3 or the stimulation of its expression needs precise mechanistic exploration.

## B7-H4

The genomic DNA of human coinhibitor B7-H4 (also referred to as B7x, B7S1, or V−set domain containing T-cell activation inhibitor 1) has been mapped to human chromosome 1p11.1, whereas a pseudogene of human B7-H4 has been mapped to chromosome 20p11.1. At the amino acid level, human B7-H4 shares its highest homology (31%) with B7-H3 and 24–31% homology with other members of the B7 family. B7-H4 inhibits T cells via cell cycle arrest and the inhibition of cytokine production, but its corresponding receptor remains unknown. Although B7-H4 mRNA is widely distributed, B7-H4 protein shows a limited distribution and is not detectable in most healthy human tissues (83). B7−H4 is a distinctive surface marker on M2 macrophages. The coculture of human placental mesenchymal stem cells (pMSCs) with monocytes stimulates M2-like differentiation and B7-H4 elevation, which suggests the involvement of B7-H4 in the immunoregulatory effect (84). Interestingly, the percentages of decidual B7-H4-positive cells in the subpopulation of CD14^+^ cells do not differ among tissue samples derived from patients who have undergone cesareans at different stages of labor (85). Alterations in macrophage activity, however, have been documented. The constant percentage of B7-H4-positive cells might account for the restriction of immune cell action that is initiated as the labor homeostatic mechanism in the reproductive tract is set in motion. The development of immune tolerance during pregnancy thus appears to require a combination of regulatory mechanisms. The expression of B7-H4 is significantly higher on CD1c^+^ myeloid and CD303^+^ plasmacytoid DCs of pregnant women at the first trimester than in pregnant women at the luteal phase of the ovarian cycle, whereas no significant difference was found in the second and third trimesters. This transient higher expression of B7-H4 suggests the role it plays in immunomodulation during early pregnancy (86). Compared with healthy pregnancy, the expression of B7-H4 on myeloid and plasmacytoid DCs is higher on CD1c^+^ myeloid DCs of patients with preeclampsia than on those of patients without preeclampsia. The B7-H4 molecule on myeloid DCs might be the tolerogenic mechanism secondary to the proinflammatory response that is observed in preeclampsia. Further studies have shown that the soluble B7−H4 (sB7−H4) blood serum concentration is elevated in both the first and third trimesters in patients with preeclampsia (87). The significantly higher level of sB7-H4 in the first trimester has some predictive ability to identify patients with an elevated risk of developing preeclampsia. In the third trimester, the serum levels of sB7-H4 and B7-H4 mRNA expression are highest in the placenta not only in early-onset PE but also in late-onset preeclampsia. A significantly high sB7-H4 level is detected in patients who develop preterm premature rupture of the amniotic membrane (pPROM). Increased serum levels of sB7-H4 in pPROM patients indicate the dynamics of the immune response at the maternal–fetal interface and might also serve as a predictive biomarker for discriminating pPROM patients and healthy controls (88). Additionally, the sB7-H4 blood serum concentrations on the day after delivery and during the peripartal phase are comparable; however, the concentration level is higher in women who experience spontaneous-onset labor than in those who undergo elective cesarean section, which indicates that sB7-H4 is involved in the maintenance of postdelivery homeostasis (89). Despite the limited knowledge of B7-H4, the available evidence strongly suggests its involvement in several pregnancy complications, which indicates that this molecule might still serve as a competitive marker for the identification of morbid pregnancy complications.

## B7-H5

The V-domain Ig suppressor of T-cell activation (VISTA), which is also known as B7-H5 and shares 24% identity with the CD80 extracellular domain, has been mapped to human chromosome 10q22.1. VISTA is encoded by Vsir in mice and c10 or f54 in humans and is predominantly expressed on hematopoietic cells, particularly within the CD11b^hi^CD14^dim^ CD16^+^/CD11b^hi^CD14^+^CD16^±^ blood monocyte population and both the lymphoid CD11^lo^CD123^+^HLA-DR^+^ and myeloid CD11c^+^CD123^lo^HLA-DR^+^ subsets of DCs (90). VISTA is also highly expressed on naïve Tregs. Although its binding chaperone has not been identified, VISTA can function as both a ligand and a receptor to conduct coinhibitory signals to suppress T-cell activation, proliferation, and cytokine production (90, 91). Other studies have revealed that VISTA interacts with V-set and Ig domain-containing 3 (VSIG3). Despite its high expression in placental tissue and potent induction of fetal tolerance, more focus has been placed on the role of VISTA in antitumor immunity. VISTA is involved in the regulation of Th2 cell differentiation. It serves as a potent suppressor of T-cell activation and upregulates the expression of Foxp3 to prevent GVHD in a mouse model (92). Targeting VISTA can offer a new approach for boosting the response rate to immunotherapy. Because normal pregnancy favors a relatively immunosuppressive environment to tolerate fetal antigens, VISTA in decidual immune cells can regulate maternal fetal immunity. The immune checkpoint therapy of tumors has revealed a potential relationship between VISTA and PD-L1. These two molecules show not only similar homology but also consistent expression after treatment with ipilimumab (93). This relationship provides a new perspective for investigating and treating PD-L1-related pregnancy complications.

## Other Members of the B7 Family: BTNL2, B7-H6, and B7-H7

Butyrophilin-like 2 (BTNL2) is a butyrophilin family member that lacks the prototypical B30.2 ring domain. BTNL2 shares 24% homology with the B7 family and localizes to human chromosome 6p21.32. With its putative receptor, which is not CD28, CTLA4, ICOS, or PD-1, BTNL2-Ig inhibits T-cell proliferation and is involved in multiple autoimmune diseases, such as sarcoidosis and myositis (94, 95). B7-H6 is a ligand for NKp30, a member of the CD28 family that functions as an NK-activating receptor. Localized on human chromosome 11p15.1, B7-H6 is undetectable at both the protein and transcriptional levels in normal tissues and unstimulated healthy peripheral blood mononuclear cells and is regarded as a new weakness of tumors (96). Human endogenous retrovirus-H long terminal repeat-associating protein 2 (HHLA2, also known as B7-H7) uniquely maps to human chromosome 3q13.13 and has 10% homology with the B7 family. Transmembrane and immunoglobulin domain-containing 2 (TMIGD2) serves as the receptor for HHLA2, and the level of this protein is limited in normal tissues but high in many malignant carcinomas (97, 98). No consolidated data have shown a significant role for these newly found members in pregnancy or its complications. Unlike B7-H6 and B7-H7, which are devoid of expression in pregnancy-related tissues based on current knowledge, BTNL2 is likely an element of maternal–fetal tolerance. Recombinant BTNL2-Ig is now regarded as a potential treatment for patients with graft-versus-host disease (99). Another study has shown that recombinant mouse BTNL2 can modify B7/CD28 signaling to promote the generation of Tregs, and these induced Tregs have basically the same immunophenotype and gene profile as natural Tregs (100). The role of BTNL2 in dictating T-cell differentiation and promoting dominant tolerance provides insights into how inhibitory signaling can mediate the immune status at the maternal–fetal interface.

## Discussion

**FIGURE 3:**
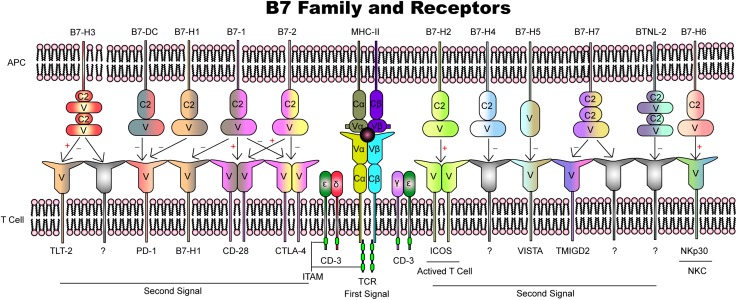
Overview of B7 family members and their receptors.

Maternal–fetal immunity is predominantly a T-cell signaling molecule-dependent process involving APCs and effector cells that results in the recognition and tolerance of semi-allogeneic fetuses from insemination to delivery. Based on expression and functional analyses, B7 family members play vitally important roles in the regulation of cell proliferation and differentiation and cytokine secretion (Figure 3 and Table 1). Among all the molecules identified, B7-1, B7-2, B7-H1, B7-DC, and B7-H2 have been well characterized and play a relatively clear role in pregnancy, although their interactions and modulation networks remain unknown. B7-H3, B7-H4, and B7-H5 share low identity with the B7 family, and little focus has been placed on their roles at the maternal–fetal interface. However, the available evidence shows that PD-L1, B7-H3, and B7-H5 are highly expressed in gestational trophoblastic neoplasia, indicating their potential functions. BTNL2, B7-H6, and HHLA2 are newly found members but play markedly more insignificant roles in pregnancy and its complications. Single-cell sequencing has profiled the individual cell types and their transcriptomes, and we believe that a more in-depth view of these molecules is needed.

**TABLE 1:** Overview of B7 family members.

	Alternative name	Genetic homology	Human chromosome	Receptor(s)	Function(s)
B7-1	CD80	100%	3q13	CTLA-4/CD28	Costimulatory Promote T-cell proliferation Stimulate Th1 response Increase IL-2, IL-3, and IFN-γ secretion Prolong effector cell lifespan
B7-2	CD86	27%	3q21	CTLA-4/CD28	Coinhibitory: Inhibit T-cell proliferation Stimulate Th2 response
B7-H1	CD274/PD-L1	25%	9p24	PD-1	Inhibit T-cell proliferation Decrease IL-2, IL-10, and IFN-γ secretion Increase TNF-α secretion Promote CTL apoptosis
B7-DC	CD273/PD-L2	23%	9p24	PD-1	Inhibit T-cell proliferation Increase IFN-γ secretion
B7-H2	ICOSL/B7RP-1/ CD275/GL50	20%	21q22	ICOS	Promote T-cell proliferation Increase IL-4, IL-5, IL-10, IFN-γ, and TNF-α secretion Stimulate Th2 response
B7-H3	CD276/B7RP-2	20%	15q24	TLT-2/?	Costimulatory: Promote T-cell proliferation Stimulate Th1 response Increase IFN-γ secretion Activate CTLs Coinhibitory: Inhibit T-cell proliferation Stimulate Th2 response Decrease IL-2 and IFN-γ secretion
B7-H4	B7x/B7S1	24%	1p13.1	?	Inhibit T-cell proliferation Decrease IL-2, IL-17, and IFN-γ secretion Inactivate CTLs
B7-H5	VISTA	24%	10q22.1	VISTA	Inhibit T-cell proliferation Increase IL-6, IL-8, IL-1β, and TNFα secretion
BTLN2	BTL-II/BTN7	24%	6p21.32	?	Inhibit T-cell proliferation
B7-H6	NCR3LG1	10%	11p15.1	NKp30	Induce NK activation
B7-H7	HHLA2	10%	3q13.13	TMIGD2/?	Inhibit T-cell proliferation Decrease IFN-γ, TNF-α, IL-2, IL-5, IL-10, IL-13, IL-17A, and IL-22 secretion

Despite its therapeutic potency for human malignancies, the blockade of immune checkpoint B7 family members, e.g., B7-H1-PD1, has a promising outcome in pregnancy and its complications. B7-H1 expression endows Tregs with an enhanced function in suppressing detrimental autoimmune disease and anti-inflammation. Therefore, B7-H1 agonists will be a critical therapeutic avenue. A novel chimeric antigen receptor (CAR) coexpressing a B7-H2-41BB fusion protein significantly promotes CAR–T-cell proliferation *in vitro* and might aid in the rejection of solid tumors (101). Other members of the B7 family, however, have been partially employed in transplantation immunity but not in maternal–fetal immunity. Early results from a pilot study showed that CTLA4 Ig-primed donor lymphocyte infusion (DLI) treatment results in progression-free survival in refractory aggressive B-cell lymphoma patients. One hypothesis is that CTLA4 Ig-DLI blocks the CD28–CD86 prosurvival pathway (102). Because the current therapies for recurrent miscarriage, including anticoagulant drugs, immunosuppressive agents, hormones, and even intravenous immunoglobulin, do not provide a stable positive pregnancy outcome, cosignaling molecules and immune checkpoints might become a promising solution, but further work is needed to widen this research area.

## Author Contributions

YZ participated in the design, execution, and analyses of the review and drafted the manuscript. QZ and LJ supervised the project, participated in critical discussions, and revised the manuscript.

## Conflict of Interest

The authors declare that the research was conducted in the absence of any commercial or financial relationships that could be construed as a potential conflict of interest.
